# Association between Schizophrenia and Violence: The Cage of Psychosis

**DOI:** 10.1192/j.eurpsy.2025.2207

**Published:** 2025-08-26

**Authors:** A. Castelao Escobar, C. Peláez Fernández, P. Sanz Sánchez, R. González Nuñez, C. Alguacil Núñez, R. Albillos Pérez

**Affiliations:** 1 Psiquiatría, Hospital Universitario Puerta de Hierro Majadahonda; 2 Psiquiatría, Hospital Universitario El Escorial, Madrid, Spain

## Abstract

**Introduction:**

In Spain, approximately 4% of the prison population suffers from severe mental disorders, with schizophrenia being more prevalent in this group compared to the general population. Although violent behavior is infrequent among individuals with schizophrenia, it holds significant clinical importance. Reoffending rates are low, and crimes are typically less severe. Comorbid substance abuse is also common.

**Objectives:**

This study examines the case of a man with schizophrenia who committed homicide under the influence of drugs, highlighting the complex relationship between schizophrenia and violence.

**Methods:**

We present the case of a 30-year-old man with no prior medical or legal history, sentenced to five years and nine months in prison for homicide (of his sister), resisting arrest, and minor injuries. The homicide occurred after the consumption of MDMA. The initial forensic report revealed no severe psychopathy and a preserved sense of reality, while the defense argued moderate impairment of cognitive and volitional faculties due to intoxication and extreme fatigue.

**Results:**

After spending a few months in prison, he attempted suicide by hanging, which did not require admission and was considered reactive to a stressful life situation. During his incarceration, the patient exhibited progressive thought disorganization, eccentric behaviors, hallucinations, and delusional ideation unrelated to substance use. He also engaged in severe self-harm, requiring bilateral orchiectomy. Following this, he was diagnosed with schizophrenia and treated in a psychiatric unit. Since 2022, he has been included in the Integrated Care Program for Severe Mental Illnesses in Prison (PAIEM) and treated with extended-release paliperidone. After release, he was incorporated into the Continuity of Care Program, maintaining regular consultations with psychiatry, nursing, and social work. He has integrated well into the psychosocial rehabilitation center, showing no behavioral disturbances. He reports almost complete amnesia of the offenses for which he was convicted and exhibits some indifference towards them. He continues to receive treatment and is diagnosed with schizophrenia, predominantly with negative symptoms.

**Conclusions:**

This case underscores the need to adequately assess negative symptoms of schizophrenia and their impact on violent behavior. The successful transition of the patient from prison to a specialized center highlights the importance of continuous treatment and monitoring in cases of severe mental disorders. Effective management of treatment and post-prison follow-up is crucial for minimizing risks and promoting successful community integration.

**Disclosure of Interest:**

None Declared

